# Individualized Prediction of Changes in 6-Minute Walk Distance for Patients with Duchenne Muscular Dystrophy

**DOI:** 10.1371/journal.pone.0164684

**Published:** 2016-10-13

**Authors:** Nathalie Goemans, Marleen vanden Hauwe, James Signorovitch, Elyse Swallow, Jinlin Song, Collaborative Trajectory Analysis Project (cTAP)

**Affiliations:** 1 University Hospitals Leuven, Child Neurology, Leuven, Belgium; 2 Analysis Group, Inc., 111 Huntington Ave, 14^th^ floor, Boston, Massachusetts, United States of America; 3 The Trajectory Analysis Project (TAP) Collaboration, One Broadway, 14^th^ floor, Cambridge, Massachusetts, United States of America; Cincinnati Children's Hospital Medical Center, UNITED STATES

## Abstract

**Background:**

Deficits in ambulatory function progress at heterogeneous rates among individuals with Duchenne muscular dystrophy (DMD). The resulting inherent variability in ambulatory outcomes has complicated the design of drug efficacy trials and clouded the interpretation of trial results. We developed a prediction model for 1-year change in the six minute walk distance (6MWD) among DMD patients, and compared its predictive value to that of commonly used prognostic factors (age, baseline 6MWD, and steroid use).

**Methods:**

Natural history data were collected from DMD patients at routine follow up visits approximately every 6 months over the course of 2–5 years. Assessments included ambulatory function and steroid use. The annualized change in 6MWD (Δ6MWD) was studied between all pairs of visits separated by 8–16 months. Prediction models were developed using multivariable regression for repeated measures, and evaluated using cross-validation.

**Results:**

Among n = 191 follow-up intervals (n = 39 boys), mean starting age was 9.4 years, mean starting 6MWD was 351.8 meters, and 75% had received steroids for at least one year. Over the subsequent 8–16 months, mean Δ6MWD was -37.0 meters with a standard deviation (SD) of 93.7 meters. Predictions based on a composite of age, baseline 6MWD, and steroid use explained 28% of variation in Δ6MWD (R^2^ = 0.28, residual SD = 79.4 meters). A broadened prognostic model, adding timed 10-meter walk/run, 4-stair climb, and rise from supine, as well as height and weight, significantly improved prediction, explaining 59% of variation in Δ6MWD after cross-validation (R^2^ = 0.59, residual SD = 59.7 meters).

**Conclusions:**

A prognostic model incorporating timed function tests significantly improved prediction of 1-year changes in 6MWD. Explained variation was more than doubled compared to predictions based only on age, baseline 6MWD, and steroid use. There is significant potential for composite prognostic models to inform DMD clinical trials and clinical practice.

## Introduction

Approximately one in every 3,600 live male births is affected by Duchenne muscular dystrophy (DMD), an inherited, X-linked disease caused by mutations to the gene encoding dystrophin [1, 2]. Limitations in ambulatory function are among the earliest signs of the disease, and are progressive. Patients initially develop muscle strength and ambulatory ability during their early childhood years, but face increasing deficits relative to normal function as they age, and eventually experience accelerating declines. Loss of independent ambulation usually occurs by the middle teens. Cardiac and pulmonary function are also impacted, contributing to a median survival of approximately 25 years [3, 4]

Significant drug development efforts in DMD have been directed towards slowing the rate of decline in ambulatory function [5–8]. The primary ambulatory outcome measure in recent clinical trials has been the distance walked in six minutes (6MWD). To date, however, clinical trials have been complicated by high levels of 6MWD variability. Within a 48-week trial period, for example, changes in 6MWD among individual patients have spanned the full range from improvement to complete loss of function [5]. There is also the potential for sub-maximal performance of the 6MWD test to occur at baseline or at follow-up assessments. This variability has confounded the accurate measurement of treatment effects, despite efforts to homogenize trial enrollment based on age and baseline 6MWD. Natural history studies have corroborated the levels of 6MWD variation observed in clinical trials [9]. Importantly, variability in 6MWD across DMD patients is attributable primarily to biological variation in rates of progression. Although 6MWD is effort based, and can be influenced by motivation, test-retest reliability is high, with correlations exceeding 0.9 [10]. An improved understanding of the factors associated with biological variation in rates of 6MWD progression will be important for continued design of clinical trials and measurement of drug effects in DMD.

To this end, prognostic factors for changes in 6MWD have been studied in DMD. Age and baseline 6MWD, in particular, have been associated with rates of 6MWD progression [9]. Steroid use has also been associated with preservation of ambulatory function in clinical trials and in retrospective analyses of long-term observational data [11]. As such, these three factors: age, baseline 6MWD and steroid use, have constituted the conventional patient characteristics used in the design of DMD clinical trials measuring drug effects on 6MWD. Inclusion criteria, stratified randomization procedures and subgroup pre-specifications, for example, have been based primarily on these three characteristics [2, 8]. Despite these efforts, significant levels of unexplained variability in 6MWD outcomes have persisted, with standard deviations for changes in 6MWD typically exceeding twice the size of the treatment effect that trials are designed to detect. This has complicated the interpretation of drug effects across multiple studies [12].

Beyond age, baseline 6MWD and steroid use, a number of functional measures have been individually or anecdotally associated with changes in 6MWD [10, 13, 14]. Multiple timed functions tests, in particular, are routinely measured in clinical practice and in clinical trials. This wealth of readily available functional measures presents potential opportunities to improve prognosis. In particular, combining multiple measures into a composite score might improve prognostic accuracy in DMD beyond that already provided by age, baseline 6MWD and steroid use [15]. Composite prognostic scores have been useful for clinical trial design and clinical practice across multiple other therapeutic areas [16–18]. In drug efficacy trials, prognostic scores have been incorporated into inclusion criteria and stratification criteria to manage variability and reduce sample size requirements [18–20]. Prognostic scores have also been used to define patient populations that benefit from treatment [21]. Comparisons of treated patients to natural history controls have used prognostic scores to define efficient matching criteria, particularly for small sample sizes [8, 22, 23]. As DMD is a rare disease with rates of progression that are highly heterogeneous compared to the effect sizes hypothesized for recently studied drug treatments, improved prognosis—i.e., explaining heterogeneity in disease progression—could significantly improve clinical trial design and analyses of drug efficacy.

With these ultimate aims in mind, we evaluated whether a combination of multiple, easily measured patient characteristics could be used to improve 1-year prognosis for changes in 6MWD.

## Study Design and Methods

### Patients

Natural history data were collected from boys aged 4.4 years or older diagnosed with DMD, monitored in routine clinical practice at the Universitaire Ziekenhuizen (UZ) Leuven pediatric neurology clinic in Leuven, Belgium. Assessments of 6MWD occurred approximately every 6 months, and included concurrent assessments of height, weight, steroid use and timed function tests. Dystrophin genotypes were also recorded. Data collection for the present natural history study spanned October 2008 to November 2015, and was truncated for patients entering clinical trials.

**Fig 1:**
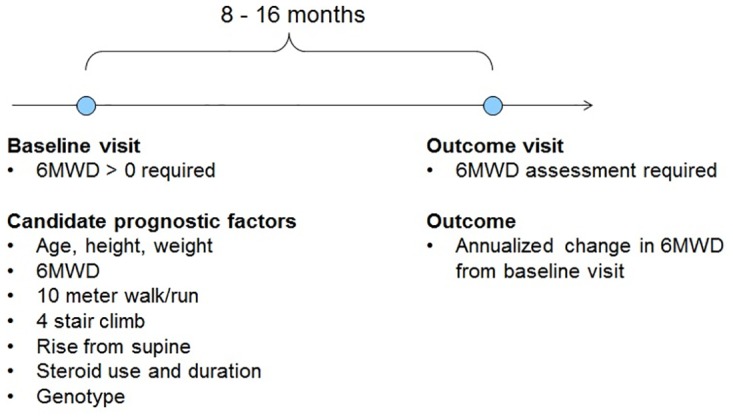
Study design. Changes in 6MWD were studied over approximate 1-year follow-up intervals (8–16 months). If a patient had multiple qualifying intervals, all such intervals were included in the analyses. 6MWD = six-minute walk distance.

To be included in the present analysis, boys were required to have at least one visit with 6MWD > 0 and at least one additional follow-up assessment of 6MWD. Changes in 6MWD were then studied for all pairs of 6MWD assessments separated by approximately one year. In particular, the study included all pairs of 6MWD assessments meeting the following criteria: 1) 6MWD > 0 at the first visit in the pair (defined as the baseline visit), 2) at least one subsequent 6MWD assessment after 8–16 months, and 3) all baseline measures non-missing. If a patient had multiple pairs of visits meeting the above criteria, all such pairs were included in the analyses (Fig 1).

Baseline measures included age, height, weight, timed rise from supine, timed 10 meter walk/run, timed 4 stair climb, 6MWD, and steroid use history. Timed function tests and all assessments were performed by trained clinical staff at UZ Leuven. Dystrophin genotypes were also assessed, and were classified in the present study as deletions, duplications, point mutations, or other alterations. Previous analyses of this cohort, based on an earlier data cut, have been published [24].

This study was approved by the Ethics Committee of the University Hospitals Leuven, and was conducted according to the Declaration of Helsinki. Written consent was obtained from the parents of all boys to report their clinical assessment data anonymously in this observational study.

### Statistical methods

Baseline characteristics were described using means and standard deviations for continuous measures; counts and percentages were used for categorical measures. The primary outcome measure, annualized change in 6MWD (Δ6MWD), was defined for each approximate 1-year interval (8–16 months) as the absolute change in 6MWD between assessments divided by the corresponding number of elapsed years. Pearson correlations were studied between Δ6MWD and each baseline characteristic, and for each pair of baseline characteristics.

Multivariable linear regression models were used to study associations between Δ6MWD and baseline characteristics. An initial model (Model 1) included only the conventional prognostic factors: age, baseline 6MWD and steroid (dichotomized as duration of use greater than 1 year at the time of the baseline visit). A second, broader, model (Model 2) included the aforementioned predictors in addition to weight, height and the timed function tests (TFTs). TFT results were included in the model as separate effects for the ability to complete the test (as a binary yes/no measure) and the time to completion (as a continuous measure set to 0 among patients who could not complete the test). A third model assessed the addition of genotypic class to the multivariable regression in Model 2. Sensitivity analyses explored the effects of adding and removing specific variables to Models 1 and 2. Repeated measures were accommodated in all models using generalized estimating equations with an exchangeable covariance structure.

The predictive performance of these models was measured as the distance between observed and predicted values of Δ6MWD. In particular, the root mean squared error (RMSE) was computed as the standard deviation of differences between observed and predicted Δ6MWD. The R^2^ value was used to measure the proportion of variance in Δ6MWD explained by the model. Descriptive analyses of baseline characteristics and outcomes were also conducted for patients stratified by quartiles of predicted Δ6MWD. These analyses were pre-specified in a Statistical Analysis Plan developed within the TAP Collaboration.

To further assess the predictive performance and robustness of the broad multivariable model (Model 2), a cross-validation approach was applied. The model was fit to 80% of the patients (the training set: 31 patients and, on average, 153 follow-up intervals) and used to generate predictions for the remaining 20% (evaluation set: 8 patients and, on average, 38 follow-up intervals). This process was repeated for 200 random splits of the data, and RMSE results were averaged across the 200 evaluation samples. To obtain a 95% confidence interval (CI) for RMSE a bootstrapping procedure was used to resample, with replacement, from the 39 patients. For each of 1000 bootstrap iterations, a cross-validated estimate of RMSE was generated. Confidence limits were obtained from the 2.5^th^ and 97.5^th^ percentiles of the bootstrapped RMSE distribution.

Finally, to explore the contributions of different baseline characteristics to the overall prognostic value of the model, R^2^ values were estimated for a series of multivariable models obtained by separately adding each individual variable to Model 1 and by separately removing each individual variable from Model 2.

## Results

**Fig 2:**
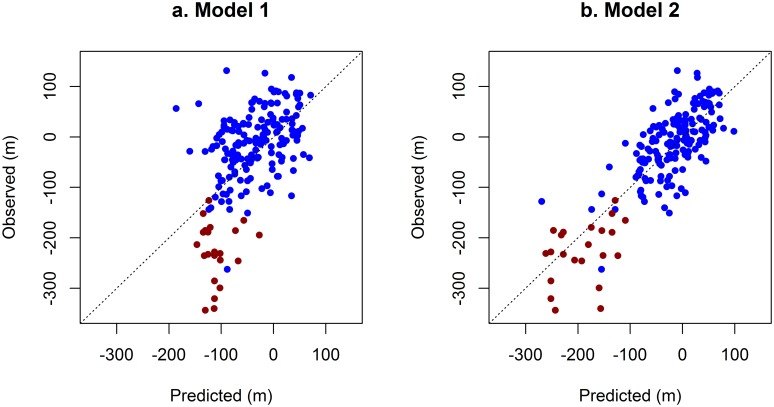
Observed vs. predicted changes in 6MWD. Predictions of Model 1 (a) and Model 2 (b). Each point represents one of the 191 follow-up intervals. Red points indicate intervals during which the patient lost the ability to complete the 6MWD test. 6MWD = six-minute walk distance; n = number; R^2^ = goodness of fit.

Among a total of 54 patients with ambulatory 6MWD assessments, 39 met the inclusion criteria and contributed a total of 191 approximate 1-year intervals of follow-up time to the analyses (Fig 2). The average number of follow-up intervals was approximately 5 per patient. The majority of the intervals were 12 ± 1 months in length.

Ages at baseline ranged from 4.4 to 15.7 years, with a mean of 9.4 years and a standard deviation (SD) of 2.4 years. Mean 6MWD at baseline was 351.8 meters (m) with a SD of 80.1 m. Steroids were being used at 86.4% (165/191) of baseline assessments; at most baseline assessments, 74.9% (143/191), patients had been using steroids for at least 1 year. All 39 patients eventually received steroids; 32 (82.1%) received deflazacort only, 5 (12.8%) received prednisone only, 1 (2.6%) switched from deflazacort to prednisone, and 1 (2.6%) switched from prednisone to deflazacort. At assessments without ≥ 1 year of prior steroid use, the average age was two years younger than at other visits (7.9 vs. 9.9 years). Among patients who could complete the timed function tests, the median (interquartile-range) was 4.4 (3.1 to 6.6) seconds for rise from supine (84.8% able to complete), 2.8 (2.0 to 4.8) seconds for the timed 4 stair climb (94.2% able to complete), and 5.2 (4.2 to 6.8) seconds for the 10 meter walk/run (all patients able to complete). The maximum observed times to completion were 24, 21, and 15 seconds, respectively, for the rise from supine, 4 stair climb, and 10 meter walk/run tests.

The mean Δ6MWD, i.e. the mean annualized change from baseline, was—37.0 m with a SD of 93.7 m (median = -16.3; range -343.7 to 131.4). Among the 191 study intervals, the ability to complete the 6MWD test was lost in 24 cases (12.6%).

Pearson correlations were used to determine the strength of association between patient’s baseline characteristics and subsequent 1 year changes in 6WMD (Table 1a). All studied baseline characteristics had statistically significant associations with Δ6MWD. Moderately positive associations were observed between declines in 6MWD and older age, lower baseline 6MWD and longer duration of steroid use (magnitudes from 0.3 to 0.5; all statistically significant). Associations were stronger for the timed rise from supine, the timed 4-stair climb, and the timed 10 meter walk/run; longer times to complete these tests at baseline were associated with greater subsequent declines in 6MWD (magnitudes of correlation > 0.6; all statistically significant). Greater height and weight were also significantly associated with greater declines in 6MWD. Δ6MWD did not differ significantly across genotypic classes (p = 0.554), with averages of -34.8, -52.3, and -20.1 m, for patients with deletions, duplications, and point mutations, respectively.

**Table 1:** Correlations between each baseline characteristic and a) 1-year change in 6MWD and b) other baseline characteristics.

	Baseline Characteristics								
	Age	Steroids^†^	Height	Weight	BMI	6MWD	10MWR	Rise^‡^	4SC^‡^
*a) Associations with 1-year change in 6MWD*									
Δ6MWD	-0.50	-0.45	-0.47	-0.40	-0.30	0.33	-0.62	-0.67	-0.65
*b) Associations with other baseline characteristics*									
Age	-	0.63	0.79	0.78	0.65	-0.25	0.54	0.46	0.39
Steroids^†^	-	-	0.30	0.34	0.38	-0.18	0.39	0.30	0.37
Height	-	-	-	0.87	0.59	-0.13	0.46	0.49	0.37
Weight	-	-	-	-	0.90	-0.25	0.52	0.48	0.42
BMI	-	-	-	-	-	-0.28	0.46	0.36	0.36
6MWD	-	-	-	-	-	-	-0.75	-0.63	-0.64
10MWR	-	-	-	-	-	-	-	0.74	0.71
Rise^‡^	-	-	-	-	-	-	-	-	0.79

Caption: All correlations were statistically significant (p<0.05). 6MWD = six-minute walk distance; Δ6MWD = annualized change in six-minute walk distance; 10MWR = ten-meter walk/run; BMI = body mass index; 4SC = four-stair climb; rise = rise from supine.

^†^Duration of use

^‡^For the purposes of these correlation analyses, patients who could not complete a test in < 30 seconds were assigned a value of 30 seconds.

Associations were also studied for each pair of potential prognostic factors. Significant correlations were also observed among all pairs of baseline characteristics (Table 1b). Age was positively associated with steroid duration, height and weight (correlation coefficients > 0.6), and with the timed function tests (0.39 to 0.54). The negative association between age and 6MWD was smaller in magnitude (0.25). All functional tests were strongly correlated with each other (magnitudes 0.63 to 0.79).

When multiple baseline characteristics were combined to predict Δ6MWD, Model 1, including only age, baseline 6MWD, and steroid use as predictors, explained 28% of the variation in Δ6MWD (Table 2a). Only age was a statistically significant predictor in this model, with older age associated with a higher rate of decline in 6MWD. Unexplained variation in Δ6MWD, as measured by the RMSE, was 79.4 m in this model (Fig 2a).

**Table 2:** Comparison of Multivariable models for annualized change in 6MWD.

	a. Model 1		b. Model 2	
	6MWD, age, steroid predictors *(RMSE = 79*.*4 m*, *R*^*2*^ *= 0*.*28)*		A broader set of predictors *(RMSE = 52*.*3 m*, *R*^*2*^ *= 0*.*69)*	
Baseline characteristics	Coefficient	95% CI	Coefficient	95% CI
Intercept	135.8	(24.8, 246.7)*	812.5	(274.1, 1350.9)**
Age, years	-21.1	(-30.9, -11.2)***	-2.4	(-7.9, 3.2)
Steroids ≥ 1 year	-15.4	(-40.0, 9.2)	-24.8	(-43.0, -6.6)**
6MWD, m	0.09	(-0.12, 0.29)	-0.48	(-0.64, -0.32)***
Height, cm			-6.8	(-11.3, -2.3)**
Weight, kg			12.9	(4.9, 21.0)**
BMI, kg/m^2^			-17.1	(-29.6, -4.6)**
Time to walk/run 10 m, s			-10.0	(-17.3, -2.8)**
Able to rise from supine			60.4	(-12.6, 133.5)
Time to rise from supine, s^†^			-4.2	(-7.4, -1.0)*
Able to climb 4 stairs			224.9	(139.9, 309.9)***
Time to climb 4 stairs, s^†^			-12.9	(-20.7, -5.2)**

Caption: 6MWD = six-minute walk distance; BMI = body mass index; RMSE = root mean squared error; R^2^ = goodness of fit; s = seconds; m = meters; cm = centimeters; kg = kilograms; CI = confidence interval.

*P < 0.05;

**P < 0.01;

***P < 0.001.

^†^Set to 0 among patients unable to complete the test.

The broader multivariable prediction model (Model 2) based on the conventional predictors plus height, weight, body mass index (BMI), and timed function tests, performed significantly better than Model 1 (Fig 2b). Model 2 accounted for almost 70% of the variation in 6MWD changes (compared to 28% in Model 1), and reduced the unexplained variation to RMSE = 52.3 m (compared to 79.4m in Model 1) (Table 2b). All baseline measures showed significant associations with Δ6MWD except for age. After cross-validation was used to obtain a conservative estimate of prognostic performance, Model 2 had an RMSE of 59.7 m (95% CI: 48.4 to 67.3) and a corresponding R^2^ of 0.59, and therefore continued to explain over twice the variation in Δ6MWD than the model based only on conventional prognostic factors.

The addition of dystrophin genotype classes (point mutation, deletion, duplication, or other) to Model 2 did not significantly improve the RMSE or R^2^ (51.7 m and 0.70, respectively), and none of the genotypic classes exhibited a statistically significant association with Δ6MWD after accounting for all of the other characteristics included in the model (data not shown). A supplementary table (S1 Table) describes the performance of a series of multivariable models obtained by adding or removing individual baseline characteristics from the analyses. The variables that reduced explained variation (R^2^) the most if removed from Model 2 were baseline 6MWD and stair climb. The variables that contributed the most when added, separately, to Model 1 were rise from supine and stair climb, followed closely by 10 meter walk/run. The variables that performed best in isolation were rise from supine and stair climb followed closely by 10 meter walk/run.

**Fig 3:**
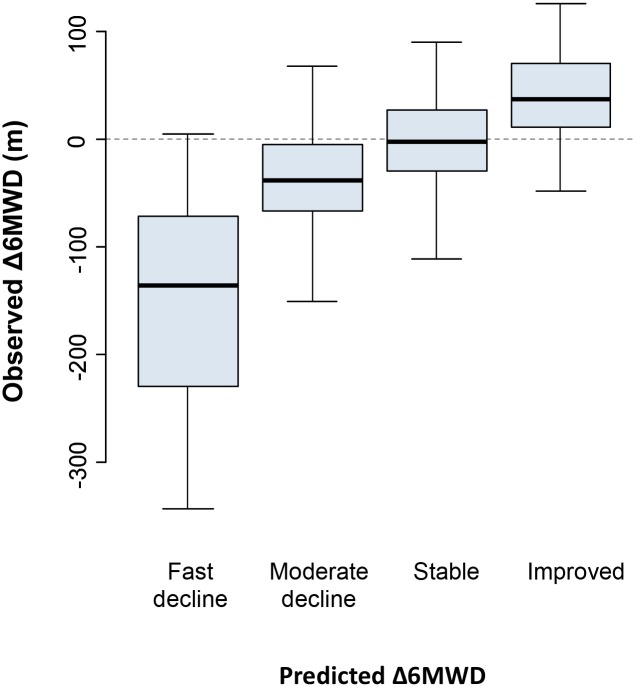
Observed changes in 6MWD stratified by baseline prediction quartiles. Solid horizontal lines indicate medians and shaded boxes indicate interquartile ranges. Vertical lines encompass the ranges of values in all groups except for the “stable” group, where the vertical lines cover 1.5 times the interquartile range and exclude two outlying values (not shown) at -116.8 and 131.4 meters. 6MWD = six minute walk distance; Δ6MWD = annualized change in six meter walk distance; m = meters.

To further characterize the predictions based on Model 2, patients were stratified into four groups based on quartiles of predicted Δ6MWD. Observed Δ6MWD differed significantly across these groups (Fig 3), and corresponded to the descriptors “fast decline,” “moderate decline,” “stable,” and “improved.” Means ± SD for observed Δ6MWD across these quartiles were -149.6 ± 92.9, -36.9 ± 48.0, -2.2 ± 50.7, and 41.2 ± 40.9 m respectively. The “fast decline” quartile included all of the patients who had lost the ability to complete the 6MWD test during the outcome period. Average baseline characteristics differed across quartiles: patients in the “fast decline” group were the oldest, with a mean age of 11.3 years (Table 3b). The “fast decline” group also had the highest rates of inability to complete the rise from supine and the 4 stair climb, and had the longest average times to completion among patients who could complete these tests. Patients aged older than 7 years were present in all quartiles. Patients with baseline 6MWD ≥ 350 meters were present in all quartiles, but were more than twice as prevalent in the middle two quartiles (“moderate decline” and “stable”) compared with the extreme quartiles.

**Table 3:** Baseline Characteristics Stratified by Predicted Change in 6MWD.

	a. Overall study sample	b. Stratification by quartile of predicted Δ6MWD*			
Baseline characteristics^†^		Fast decline	Moderate decline	Stable	Improved
	(n = 191)	(n = 48)	(n = 48)	(n = 47)	(n = 48)
Age, years	9.4 (2.4)	11.3 (1.3)	10.3 (2.0)	8.8 (2.2)	7.3 (1.7)
Age category, years					
< 5	2 (1.0)	0 (0.0)	1 (2.1)	0 (0.0)	1 (2.1)
(5, 7)	37 (19.4)	0 (0.0)	2 (4.2)	12 (25.5)	23 (47.9)
(7, 9)	44 (23.0)	2 (4.2)	7 (14.6)	15 (31.9)	20 (41.7)
(9, 12)	80 (41.9)	32 (66.7)	31 (64.6)	15 (31.9)	2 (4.2)
≥ 12	28 (14.7)	14 (29.2)	7 (14.6)	5 (10.6)	2 (4.2)
Height, cm	123.3 (10.4)	131.5 (8.1)	127.2 (8.4)	119.8 (9.0)	114.5 (6.5)
Weight, kg	29.7 (9.4)	36.1 (8.3)	32.6 (8.5)	27.6 (8.9)	22.56 (5.6)
BMI, kg/m^2^	19.1 (3.4)	20.7 (3.8)	19.8 (2.9)	18.8 (3.5)	17.0 (2.3)
Genotype					
Deletion	140 (73.3)	37 (77.1)	42 (87.5)	34 (72.3)	27 (56.2)
Duplication	36 (18.8)	9 (18.8)	5 (10.4)	8 (17.0)	14 (29.2)
Point mutation	15 (7.9)	2 (4.2)	1 (2.1)	5 (10.6)	7 (14.6)
Steroid duration, years	2.8 (2.0)	4.3 (1.8)	3.0 (2.0)	2.6 (1.9)	1.3 (1.2)
Steroid use category, months					
< 1	26 (13.6)	4 (8.3)	5 (10.4)	4 (8.5)	13 (27.1)
(1, 6)	7 (3.7)	0 (0.0)	0 (0.0)	4 (8.5)	3 (6.2)
(6, 12)	15 (7.9)	0 (0.0)	3 (6.2)	5 (10.6)	7 (14.6)
(12, 24)	29 (15.2)	1 (2.1)	9 (18.8)	8 (17.0)	11 (22.9)
(24, 60)	78 (40.8)	23 (47.9)	20 (41.7)	21 (44.7)	14 (29.2)
≥ 60	36 (18.8)	20 (41.7)	11 (22.9)	5 (10.6)	0 (0.0)
6MWD, m	351.8 (80.9)	283.8 (100.3)	398.0 (65.1)	386.0 (46.8)	339.8 (42.1)
6MWD category, m					
< 150	2 (1.0)	2 (4.2)	0 (0.0)	0 (0.0)	0 (0.0)
(150, 250)	19 (9.9)	19 (39.6)	0 (0.0)	0 (0.0)	0 (0.0)
(250, 350)	61 (31.9)	14 (29.2)	10 (20.8)	7 (14.9)	30 (62.5)
(350, 450)	89 (46.6)	11 (22.9)	25 (52.1)	36 (76.6)	17 (35.4)
≥ 450	20 (10.5)	2 (4.2)	13 (27.1)	4 (8.5)	1 (2.1)
Time to walk/run 10 m, s	5.9 (2.5)	8.9 (2.6)	5.5 (1.4)	4.8 (1.0)	4.4 (1.1)
Able to rise from supine	162 (84.8)	21 (43.8)	46 (95.8)	47 (100.0)	48 (100.0)
Time to rise from supine, s^‡^	5.8 (3.9)	11.8 (5.8)	6.4 (3.0)	4.7 (2.3)	3.6 (1.3)
Able to climb 4 stairs	180 (94.2)	37 (77.1)	48 (100.0)	47 (100.0)	48 (100.0)
Time to climb 4 stairs, s^‡^	3.9 (3.2)	8.2 (4.3)	3.4 (1.4)	2.7 (1.4)	2.2 (0.72)

Caption: 6MWD = six-minute walk distance; Δ6MWD = annualized change in six minute walk distance; n = number; s = seconds; m = meters.

*Thresholds for quartiles of predicted Δ6MWD (m) were < -73.5 for “fast decline,” [-73.5, -22.4) for “moderate decline”, [-22.4, 17.1) for “stable” and ≥ 17.1 for “improved,” and were based on Model 2.

^†^Means (standard deviations) are shown for continuous characteristics; counts (percentages) are shown for dichotomous and categorical characteristics. Intervals represented as “(,)” indicate inclusive minimum thresholds and exclusive maximum thresholds.

^‡^Calculated among the subset of patients able to complete the test.

## Discussion

In this analysis of natural history data from a single center, timed function tests provided significant prognostic value for 1-year changes in 6MWD. In particular, adding the timed 4-stair climb, rise from supine, and 10 meter walk/run, along with height and weight, to a composite prognostic model more than doubled the proportion of explained variation in Δ6MWD compared to a model based only on conventional prognostic factors (age, baseline 6MWD, and steroid use).

The composite prognostic model developed in this study builds on prior research in DMD. Associations between changes in 6MWD and individual baseline factors have been previously reported. For example, in a recent natural history study of patients with DMD, a baseline 6MWD of <350 m was associated with greater functional decline, loss of ambulation was only observed in those with a baseline 6MWD <325 m, and just 2.3% of patients able to stand from supine lost ambulation [10]. The association between age and performance on the 6MWD test has been described, with improvements occurring on average among boys younger than 7 years, and declines thereafter [25, 26]. The importance of potential composite prognostic models in DMD has been previously noted [15].

While the value of a prognostic model is most directly measured in terms of its predictive accuracy, it is important to also consider the interpretation and clinical face validity of the estimated associations driving the predictions. The estimated associations are *conditional* associations. That is, the estimated coefficients in Table 2 measure the association between a baseline characteristic and Δ6MWD when all other baseline characteristics remain unchanged. To facilitate interpretation, we consider each baseline characteristic in turn.

- **Age.** Because DMD is a progressive disease, age has been widely used as prognostic factor. In the present study, baseline age was strongly associated with Δ6MWD both individually and in Model 1. However age did not make a significant contribution to the broader Model 2. This indicates that, in the present study sample, the prognostic value of age for 1-year Δ6MWD was largely subsumed within that of height, weight, and the other functional measures, which all had strong correlations with baseline age.
- **6MWD.** When considered in isolation, baseline 6MWD was positively associated with Δ6MWD. However, the direction of this association reverses in Model 2. To help interpret this change, a scatterplot of baseline 6MWD vs. Δ6MWD is included in S2 Fig, and shows that the positive association was driven primarily by differences between boys who lose ambulation (lower baseline 6MWD and greater loss) and those who do not (higher baseline 6MWD and a mix of loss and gain). However, in S2 Fig it is apparent that within the group of boys who lose ambulation the association is necessarily negative, and, separately, within the group of boys who do not lose ambulation, the association between baseline 6MWD and Δ6MWD is weak and not obviously positive. With this in mind, it is not surprising that adjustment for additional prognostic factors reverses the association. Statistically, this is an example of Simpson’s Paradox [27]. In practical terms, it means that baseline 6MWD alone is not a reliable prognostic factor: although a small fraction of patients with extremely low baseline 6MWD are almost certain to lose ambulation in the next year, and a small fraction with very high baseline 6MWD are almost certain to maintain ambulation, patients with intermediate baseline 6MWD have a mixture of subsequent rates of change that can be best resolved by other prognostic factors. This is consistent with the presence of patients with baseline 6MWD ≥ 350 m in all quartiles when patients are stratified by predicted change in Table 3b.
- **Steroid use.** There is substantial evidence that steroid use is protective in DMD [11, 28–31]. It may therefore be surprising that steroid use is associated with greater declines in 6MWD in Model 2. The distinction must be drawn between a prognostic association (which is estimated in Model 2) and a treatment effect (which is estimated in clinical trials of steroid use). This distinction is common to all prognostic models. In the widely-used Framingham risk score for cardiovascular disease, for example, use of antihypertensive medications is associated with increased risk, even after accounting for age, blood pressure, lipids and other factors [32]. The distinction is due to non-equivalence of correlation and causation, the potential for confounding factors, and, most importantly, the fact that Δ6MWD is not studied from the time of steroid initiation. Duration of steroid use is likely to be associated with the time from first diagnosis, and the potential benefits of prior steroid use are already reflected in the baseline functional status.
- **Weight, height and BMI.** Because BMI is already a composite of height and weight, and because all of these measures are highly correlated with each other and with age, it was somewhat surprising that all three contributed statistically to Model 2. Due to the strong correlations among these measures we should not read too much into their estimated individual effects (it is not possible to significantly change one of these measures without changing the others). One observation is that higher weight versus age and height can reflect either higher muscle mass (which should enable ambulation) or higher body fat (which might limit performance on 6MWD), or some combination of the two. Our finding that height and weight, and their combination into BMI, are all important prognostic factors motivates further investigation of body composition and prognosis in DMD. Measures of lean body mass, in particular, would be informative for future analyses.
- **Timed function tests.** All three timed function tests, rise from supine, 10 meter walk/run, and 4 stair climb, showed strong associations with Δ6MWD. Longer times to complete these tests were associated with worse prognosis for 1-year Δ6MWD. In addition, inability to complete the tests, particularly the 4-stair climb, had a profoundly negative association with Δ6MWD prognosis [12]. As stated previously, the fact that these TFTs add significant prognostic information beyond that provided by baseline 6MWD, age, and steroid use is the primary finding of this study. However, it is perhaps not surprising. Physicians who see many DMD patients report developing a gestalt prognosis based largely on these measures, which have a long history of use in neuromuscular assessment [12, 33]. The composite prognostic score developed in Model 2 formalizes and quantifies this gestalt prognosis.
- **Genotypic classes.** The present study considered only broad classes of genotypes due to limited sample sizes. No significant associations were observed between Δ6MWD and any of the genotypic classes. Previous analyses of larger samples have identified associations between mutations affecting specific dystrophin exons and rates of progression in DMD [34]. As with the non-significance of age in Model 2, the effects of genotypic class on progression may already be captured by the other baseline functional measures.

Among all baseline characteristics considered in this study, which are most important for prognosis? As illustrated by the discussion of baseline 6MWD above, the prognostic importance of a variable cannot be measured in isolation. Like players in a team sport, the contribution of each must be measured in the context of others. By assessing the performance of a series of composite models, each obtained by adding and removing specific individual variables, it could be inferred that rise from supine, stair climb, 10 meter walk/run, and baseline 6MWD were the most important prognostic factors for Δ6MWD, among all of the factors considered in the present study, and that the prognostic value of baseline 6MWD was greatly enhanced when considered in conjunction with the TFTs.

Although the prognostic model developed in this study already offers potential for significant improvement over conventionally used prognostic factors (age, baseline 6MWD and steroid use), it is important to ask whether prognosis could be even further improved with additional baseline information. Magnetic resonance imaging measures of fat fraction and genetic modifiers have been associated with ambulatory outcomes in DMD, and warrant significant interest as prognostic factors [35, 36]. In addition, because multiple timed function tests were helpful for prognosis in the present analysis, it is possible that additional functional measures could bring further improvement. Measures of upper limb mobility, components of the North Star Ambulatory assessment, muscle strength testing, and other measures all warrant investigation as prognostic factors [37]. Potential associations between pulmonary and/or cardiac function and future changes in ambulatory function warrant further study, especially among older boys. Rates of decline in pulmonary function, in particular, have been previously associated with age at loss of ambulation [38]. Furthermore, due in part to the limited sample size, we did not investigate interactions among the available predictors or non-linear associations that could potentially help improve prognosis. In future investigations, it will be important to consider incremental prognostic value, i.e., the increase in prognostic accuracy when a factor is added to an already established composite score, in addition to pairwise associations with outcomes.

The prognostic model presented here is specific to the studied population and the 6MWD outcome measure. The present study included boys aged 4.4 years or older who could perform the 6MWD tests and the TFTs, and had time to manifest deficits in these measures. Prognosis in younger boys may depend more heavily on genetic factors, imaging-based biomarkers, and age-appropriate measures of strength and function [39, 40]. The 6MWD outcome was also studied over a 1-year time horizon, due to the relevance of this outcome period for clinical trials. Prognostic factors could differ for longer-term changes in 6MWD, changes in other outcome measures, or time to loss of ambulation over longer follow-up periods.

The principal limitations of this study are that natural history data were drawn from a single center and that, despite having a large number of 1-year follow-up intervals (n = 191), the number of individual patients studied was smaller (n = 39). Small sample sizes increase the risk of overfitting, which can artificially inflate measures of predictive accuracy. The use of cross-validation to obtain conservative estimates of prognostic performance, and to avoid inflation due to overfitting, was an important step towards addressing the limited sample size and demonstrating internal validity. Establishing external validity in separate data is an important future step. Evaluations in new data should consider both the quantitative prognostic performance of the model (i.e., RMSE and R^2^) and the reproducibility of the qualitative conclusions, particularly the observed prognostic importance of TFTs. This distinction between quantitative and qualitative reproducibility is important because, at the time of this study, the consistency of TFT assessments across different centers and study settings is unclear (e.g., clinical practice vs. clinical trials). The models developed in the present study used TFT results from a single center with internally consistent measurement procedures, and used the number of seconds for completion directly in the prognostic model. Transformations of TFTs (e.g., based on thresholds or conversion to velocities) could potentially improve consistency across data sources by reducing sensitivity to very long completion times, and should be further explored in the context of prognostic models. When evaluating the model in new data sources it will be important to consider the baseline comparability of the ranges of TFT measurements, and comparability in methods of assessment. We have explored model fitting with common TFT transformations in S2 Table to facilitate such comparison across databases.

A validated prognostic score is a useful tool for clinical trial design and interpretation. Once the score is calculated for each patient, it provides, by construction, a single number for each patient that has a stronger association with outcomes than any of its components. In general, a composite prognostic score can make clinical trial inclusion/exclusion, stratified randomization and matching to natural history control groups more practical and feasible compared to direct use of the multiple underlying prognostic factors, especially with small sample sizes. For example, it may be feasible to stratify randomization or match patients on the basis of a prognostic score, but not separately on multiple prognostic factors. As a rough approximation of how much value could be provided by the prognostic score described here, if reproducible in separate data, a standard power analysis (S1 Fig) shows that reducing the unexplained error in Δ6MWD from 80 m to 60 m can cut almost in half the sample size requirements for detecting treatment effect sizes that have been targeted and observed in recent clinical trials. Limiting a trial to patients with “moderate decline,” a group whose trajectories may be hypothesized to be the most modifiable within a 1-year period, could even further reduce the unexplained variability and sample size requirements. Potential increases in power, and smaller sample size requirements, could enable smaller and faster trials, and help avoid ambiguity in drug efficacy assessments in DMD.

While the impetus for the present study was to inform the design and interpretation of clinical trials, prognostic scores can also be valuable in clinical practice. For example, a validated prognostic score could potentially inform benefit-risk assessments and could help patients and families plan for loss of ambulation. The broad potential value of prognostic scores for DMD highlights the urgency for further evaluation of the score developed here.

## Supporting Information

S1 Fig Reductions in sample size requirements associated with reductions in standard deviation of Δ6MWD. Legend: Δ6MWD = change in six-minute walk distance; m = meters.
(TIF)

S2 Fig Scatterplot for baseline 6MWD vs. Δ6MWD. Legend: 6MWD = six-minute walk distance; Δ6MWD = change in six-minute walk distance; m = meters.
(TIF)

S1 Table R^2^ values after adding or removing specific baseline characteristics. Caption: *R^2^ values decreased slightly after adding certain variables; note that models were fit using generalized estimating equations which, since they account for within-patient correlation, do not necessarily minimize the marginal sum of squared prediction errors. R^2^ = measure of goodness of fit; 6MWD = six-minute walk distance; BMI = body mass index.
(DOCX)

S2 Table Fitted multivariable models incorporating different transformations of timed function tests. (DOCX)
