# Supplementary figures and images for: Individualized Prediction of Changes in 6-Minute Walk Distance for Patients with Duchenne Muscular Dystrophy
Source: PLoS One. 2016 Oct 13;11(10):e0164684. doi: 10.1371/journal.pone.0164684 (PMC5063281; doi:10.1371/journal.pone.0164684)

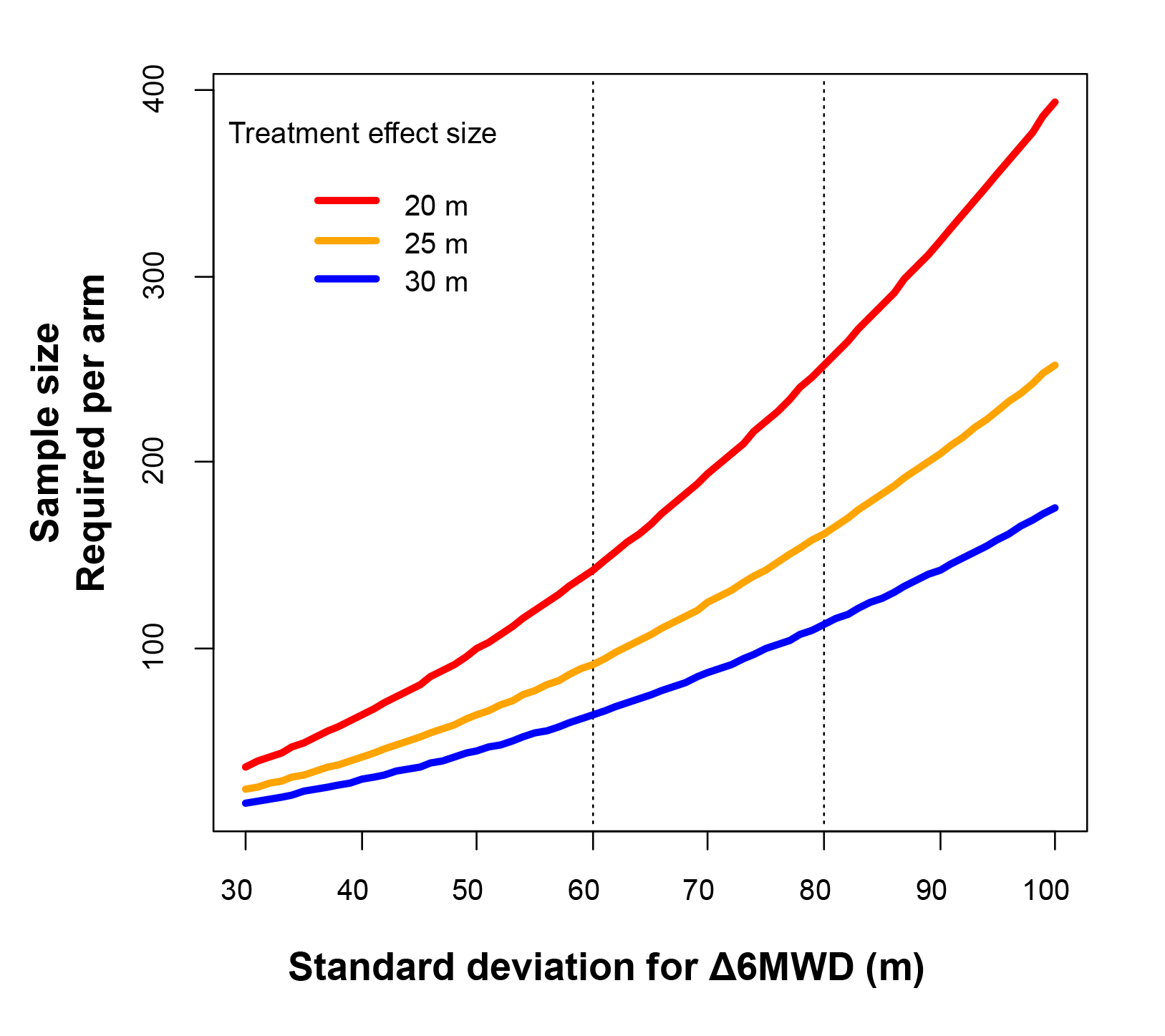

Supplement: S1 Fig — Legend: Δ6MWD = change in six-minute walk distance; m = meters. (TIF) [file pone.0164684.s001.tif]

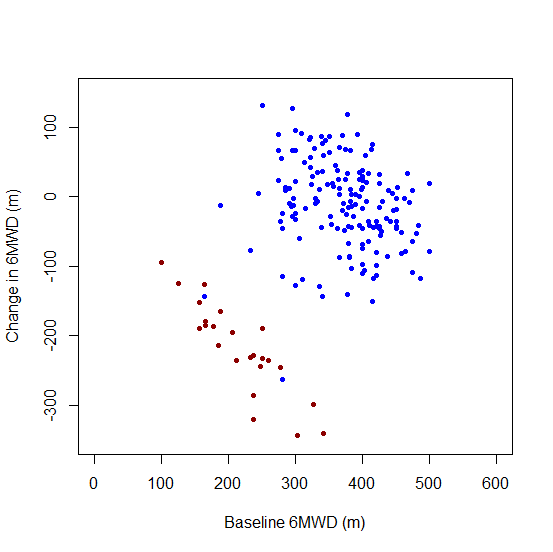

Supplement: S2 Fig — Legend: 6MWD = six-minute walk distance; Δ6MWD = change in six-minute walk distance; m = meters. (TIF) [file pone.0164684.s002.tif]
