# Supplementary material for: Individualized Prediction of Changes in 6-Minute Walk Distance for Patients with Duchenne Muscular Dystrophy
Source: PLoS One. 2016 Oct 13;11(10):e0164684. doi: 10.1371/journal.pone.0164684 (PMC5063281; doi:10.1371/journal.pone.0164684)
Supplement: S1 Table — Caption: *R2 values decreased slightly after adding certain variables; note that models were fit using generalized estimating equations which, since they account for within-patient correlation, do not necessarily minimize the marginal sum of squared prediction errors. R2 = measure of goodness of fit; 6MWD = six-minute walk distance; BMI = body mass index. (DOCX) [file pone.0164684.s003.docx]

**S1 Table. R^2^ values after adding or removing specific baseline characteristics**

| Baseline characteristics | **Removed**  **from Model 2**  (Reference = 0.69) | **Added**  **to Model 1**  (Reference = 0.28) | **Studied**  **in isolation**  (Reference = 0.00) |
| --- | --- | --- | --- |
| Age | 0.69 | 0.28 | 0.24 |
| Steroids ≥ 1 year | 0.68 | 0.28 | 0.09 |
| 6MWD | 0.63 | 0.28 | 0.09 |
| Height | 0.67 | 0.31 | 0.18 |
| Weight | 0.67 | 0.27^*^ | 0.13 |
| BMI | 0.68 | 0.27^*^ | 0.07 |
| 10 meter walk/run | 0.67 | 0.46 | 0.39 |
| Rise from supine (ability & time) | 0.68 | 0.54 | 0.47 |
| 4 stair climb (ability & time) | 0.61 | 0.61 | 0.51 |
| Genotype class | - | 0.30 | 0.00 |
