# Supplementary material for: Individualized Prediction of Changes in 6-Minute Walk Distance for Patients with Duchenne Muscular Dystrophy
Source: PLoS One. 2016 Oct 13;11(10):e0164684. doi: 10.1371/journal.pone.0164684 (PMC5063281; doi:10.1371/journal.pone.0164684)
Supplement: S2 Table — (DOCX) [file pone.0164684.s004.docx]

**Table S2: Fitted multivariable models incorporating different transformations of timed function tests**

| **a. TFT thresholds at 8 seconds** |  |  |  |
| --- | --- | --- | --- |
|  | **Estimate** | **95% CI** | **P value** |
| (Intercept) | 707.3244 | (225.6, 1189.05) | 0.00400 |
| Age (years) | -5.7339 | (-14.33, 2.86) | 0.19100 |
| Steroid use >= 1 year (yes/no) | -13.5827 | (-31.29, 4.12) | 0.13270 |
| 6MWD (m) | -0.4666 | (-0.68, -0.25) | 0.00000 |
| Height (cm) | -4.2044 | (-8.92, 0.51) | 0.08060 |
| Weight (kg) | 6.6363 | (-1.27, 14.54) | 0.10000 |
| BMI | -8.0168 | (-18.79, 2.76) | 0.14480 |
| **10MWR > 8s** | -77.1376 | (-130.18, -24.1) | 0.00440 |
| **Rise from supine > 8s** | -33.5122 | (-57.95, -9.08) | 0.00720 |
| **4SC > 8s** | -137.6379 | (-200.49, -74.78) | 0.00000 |
| *RMSE = 54.76 (m), R-squared = 0.66* |  |  |  |
|  |  |  |  |
| **b. TFT thresholds at 6 seconds** |  |  |  |
|  | **Estimate** | **95% CI** | **P value** |
| (Intercept) | 1096.9747 | (600.83, 1593.12) | 0.00000 |
| Age (years) | -5.7017 | (-16.05, 4.64) | 0.28010 |
| Steroid use >= 1 year (yes/no) | -23.1669 | (-48.14, 1.81) | 0.06900 |
| 6MWD (m) | -0.225 | (-0.46, 0.01) | 0.06360 |
| Height (cm) | -8.0208 | (-12.69, -3.35) | 0.00080 |
| Weight (kg) | 13.5996 | (5.88, 21.32) | 0.00060 |
| BMI | -18.9202 | (-31.41, -6.43) | 0.00300 |
| **10MWR > 6s** | -34.8071 | (-68.21, -1.41) | 0.04110 |
| **Rise from supine > 6s** | -7.019 | (-29.52, 15.48) | 0.54090 |
| **4SC > 6s** | -117.8384 | (-170.46, -65.22) | 0.00000 |
| *RMSE = 62.2 (m), R-squared = 0.56* |  |  |  |
|  |  |  |  |
| **c. TFT thresholds at 12 seconds** |  |  |  |
|  | **Estimate** | **95% CI** | **P value** |
| (Intercept) | 938.1376 | (511.06, 1365.21) | 0.00000 |
| Age (years) | -8.3065 | (-17.9, 1.29) | 0.08960 |
| Steroid use >= 1 year (yes/no) | -7.5845 | (-27.51, 12.34) | 0.45560 |
| 6MWD (m) | -0.3332 | (-0.55, -0.11) | 0.00280 |
| Height (cm) | -6.0304 | (-9.79, -2.27) | 0.00170 |
| Weight (kg) | 12.6775 | (4.94, 20.41) | 0.00130 |
| BMI | -19.5267 | (-32.65, -6.4) | 0.00350 |
| **10MWR > 12s** | -33.4251 | (-78.45, 11.6) | 0.14570 |
| **Rise from supine > 12s** | -105.8789 | (-151.84, -59.92) | 0.00000 |
| **4SC > 12s** | -129.9185 | (-169.96, -89.88) | 0.00000 |
| *RMSE = 58.47 (m), R-squared = 0.61* |  |  |  |
|  |  |  |  |
| **d. TFTs converted to velocities** |  |  |  |
|  | **Estimate** | **95% CI** | **P value** |
| (Intercept) | 913.9247 | (390.81, 1437.04) | 0.00060 |
| Age (years) | -4.1424 | (-13.74, 5.45) | 0.39750 |
| Steroid use >= 1 year (yes/no) | -26.6501 | (-49.28, -4.02) | 0.02100 |
| 6MWD (m) | -0.1877 | (-0.41, 0.04) | 0.10460 |
| Height (cm) | -8.4336 | (-13.2, -3.66) | 0.00050 |
| Weight (kg) | 15.7784 | (6.75, 24.81) | 0.00060 |
| BMI | -23.8588 | (-37.61, -10.11) | 0.00070 |
| **10MWR velocity (m/s)** | 9.7694 | (-13.36, 32.9) | 0.40780 |
| **Can rise from supine (yes/no)** | 62.4007 | (-9.07, 133.87) | 0.08700 |
| **Rise from supine velocity (1/s)** | 31.5614 | (-94.89, 158.01) | 0.62470 |
| **Can complete 4SC (yes/no)** | 73.3077 | (14.04, 132.58) | 0.01530 |
| **4SC velocity (1/s)** | 149.3311 | (68.31, 230.35) | 0.00030 |
| *RMSE = 61.09 (m), R-squared = 0.57* |  |  |  |
